# Empagliflozin reduces diffuse myocardial fibrosis by extracellular volume mapping: A meta-analysis of clinical studies

**DOI:** 10.3389/fendo.2022.917761

**Published:** 2022-08-11

**Authors:** Haipeng Wang, Lin Ding, Liwen Tian, Yutian Tian, Lin Liao, Junyu Zhao

**Affiliations:** ^1^ Department of Radiology, Shandong Provincial Hospital Affiliated to Shandong First Medical University, Ji’nan, China; ^2^ Department of Endocrinology and Metabology, The First Affiliated Hospital of Shandong First Medical University & Shandong Provincial Qianfoshan Hospital, Shandong Key Laboratory of Rheumatic Disease and Translational medicine, Shandong Institute of Nephrology, Jinan, China; ^3^ Department of Endocrinology and Metabology, Shandong Provincial Qianfoshan Hospital, Cheeloo College of Medicine, Shandong University, Jinan, China; ^4^ Department of Radiology, Shandong Provincial Hospital, Shandong University, Ji’nan, China

**Keywords:** empagliflozin, extracellular volume, cardiac magnetic resonance, myocardial fibrosis, meta-analysis

## Abstract

**Objective:**

The aim of the study was to evaluate the effect of empagliflozin on diffuse myocardial fibrosis by cardiac magnetic resonance (CMR) T1 mapping.

**Research methods and procedures:**

Databases including PubMed, Cochrane library, Embase, and Sinomed for clinical studies of empagliflozin on myocardial fibrosis were searched. Two authors extracted the data and evaluated study quality independently. Weighted mean difference (WMD) and 95% confidence intervals (CI) were used for continuous variables. Review Manager 5.3 was used to performed the analysis.

**Results:**

Six studies were included in this meta-analysis. One of the six studies was assessed as poor quality by the assessment of methodological quality; however, the remaining five studies were considered good. The WMD value of △extracellular volume (ECV) was merged by the fixed-effect model, and the pooled effect size was -1.48 (95% CI -1.76 to -1.21, *P* < 0.00001), which means in favor of empagliflozin. Heterogeneity analysis did not find any heterogeneity (chi^2^ = 0.39, *P* = 0.82, *I* ^2^ = 0%). In addition, empagliflozin had a tendency to reduce ECV compared to treatment before with no statistical significance (WMD = -0.29, 95% CI -1.26 to 0.67, *P* = 0.55; heterozygosity test, chi^2^ = 2.66, *P* = 0.45, *I* ^2^ = 0%). The WMD value of △native T1 was also merged by the fixed-effect model, but the pooled effect size showed neither statistical difference between empagliflozin and placebo treatment (WMD = -5.40, 95% CI -21.63 to 10.83, *P* = 0.51) nor heterogeneity (chi^2^ = 0.05, *P* = 0.83, *I* ^2^ = 0%).

**Conclusions:**

Empagliflozin has cardiovascular benefits by reducing diffuse myocardial fibrosis. ECV could act as a non-invasive imaging tool to assess diffuse myocardial fibrosis and monitor disease progression.

**Systematic Review Registration:**

https://www.crd.york.ac.uk/PROSPERO/display_record.php?RecordID=324804, identifier: CRD42022324804

## Introduction

Diffuse myocardial fibrosis has been detected in nearly all chronic cardiac diseases and characterized by diffuse interstitial and perivascular deposition of fibrotic tissue (1–5), resulting in various cardiac changes, such as adverse left ventricular remodeling, increased myocardial stiffness, and diastolic dysfunction (6). Therefore, diffuse myocardial fibrosis plays a critical role in the progression of heart failure (HF) and contributes to unfavorable outcomes in patients with HF, especially in elderly people (7–11). Notably, if the disease progression is controlled, diffuse myocardial fibrosis is potentially reversible (12, 13). Understanding the development and clinical consequences of diffuse myocardial fibrosis from a prognostic perspective is crucially important in the era of precision cardiovascular medicine (1).

Sodium-glucose cotransporter 2 (SGLT2) inhibitors, a class of antihyperglycemic drugs, are reported for their significant cardiovascular benefits (14–17). SGLT2 inhibitors have been reported to reduce the risk of major adverse cardiovascular events and hospitalization for HF in diabetic patients compared to placebo (14, 18). Meanwhile similar cardiovascular benefits to HF patients with reduced ejection fraction have also been reported, irrespective of whether they had diabetes (15, 16). SGLT2 inhibitors cause direct pleiotropic effects on the cardiac myocardium, independent of diabetic conditions (19). Furthermore, animal studies (20, 21) found that SGLT2 inhibitors could significantly improve cardiac function by reducing diffuse myocardial fibrosis. However, the underlying mechanisms leading to these cardiovascular benefits remain elusive (22, 23).

Cardiac magnetic resonance (CMR) is a mature technique for the diagnosis and evaluation of cardiac diseases. CMR not only can accurately assess the cardiac structure and function but also has a unique advantage in non-invasive myocardial tissue characterization (24–27). Extracellular volume (ECV) is a measure of the percentage of extracellular myocardium volume, which allows clinicians and researchers to quantify the extent of cardiomyopathy (28). Thus, without myocardial edema and/or inflammation, infiltration, and ischemia, ECV can unmask the cardiac pathology of diffuse myocardial fibrosis as an excellent measurement (29–31). So far, the effect of empagliflozin (a kind of SGLT2 inhibitor) on diffuse myocardial fibrosis using CMR T1 mapping for myocardial tissue characterization has been evaluated in both diabetic and non-diabetic patients (16, 32–36). Some studies demonstrated that empagliflozin could significantly improve diffuse myocardial fibrosis while others showed disagreement.

The meta-analysis was designed to investigate the effect of empagliflozin on diffuse myocardial fibrosis using CMR T1 mapping techniques.

## Materials and methods

This meta-analysis was conducted under the guidance of the Preferred Reporting Items Statement for Systematic Evaluation and Meta-Analysis (PRISMA), and it was also registered (PROSPERO ID: CRD42022324804).

### Searching progress

We searched the following databases for clinical studies of empagliflozin on ECV: PubMed, Cochrane library, Embase, and Sinomed, for clinical studies of empagliflozin on ECV. A list of references to all eligible articles and related review articles was also manually searched. A literature search of this meta-analysis was limited to published results. Databases were searched from the earliest data to 21 March 2022 with the following search terms: (“myocardial fibrosis” OR “cardiac fibrosis” OR “Myocardial Extracellular Volume” OR “extracellular volume” OR “ECV” OR “late gadolinium enhancement” OR “LGE” OR “T1 mapping” OR “T1 mapping”) AND (“SGLT2 inhibitors” OR “Sodium-Glucose Transporter 2 Inhibitors” OR “Sodium-glucose cotransporter-2 inhibitors” OR “Dapagliflozin” OR “Canagliflozin” OR “Empagliflozin” OR “Ipragliflozin” OR “Luseogliflozin” OR “Tofogliflozin”).

Eligible studies were screened and selected based on the following criteria: (1) published in English or Chinese; (2) evaluated the effect of empagliflozin on myocardial fibrosis; (3) clinical study (either cohort study or randomized control study); (4) reported at least one outcome, either ECV or native T1 value.

### Study selection and data extraction

The studies were screened independently by two authors, and any differences were resolved by consensus. If there is still doubt, a third experienced author was invited to join the consultation and reach a consensus finally. The following data were extracted from the eligible studies: (1) characteristic of populations, interventions, number of participants, mean age, male (%); (2) follow-up time; (3) MR system; (4) outcome index.

### Methodological quality assessment

The Newcastle–Ottawa Scale (NOS) was used for cohort studies and risk of bias for randomized control studies to assess the methodological quality. The score of 9 is highest for the NOS scale and shows the highest quality. For the risk-of-bias table for the randomized control trial (RCT), a point of 4 or less is considered “poor methodological quality”, while a point above 4 is defined as “good methodological quality”. Two authors scored these items independently.

### Statistical analysis

The main outcome was the change in ECV over the treatment duration. We also analyzed the change in native T1 value over the treatment duration. For continuous variables, the weighted mean difference (WMD) and 95% confidence intervals (CI) were used. The fixed-effect model was used for data analysis. *I* ^2^ was calculated as an indicator of inter-study heterogeneity. All data analysis was performed using Review Manager 5.3 (Cochrane Collaboration, United Kingdom, http://www.cochrane.org).

## Results

### Search results and characteristics of included studies

**Figure 1:**
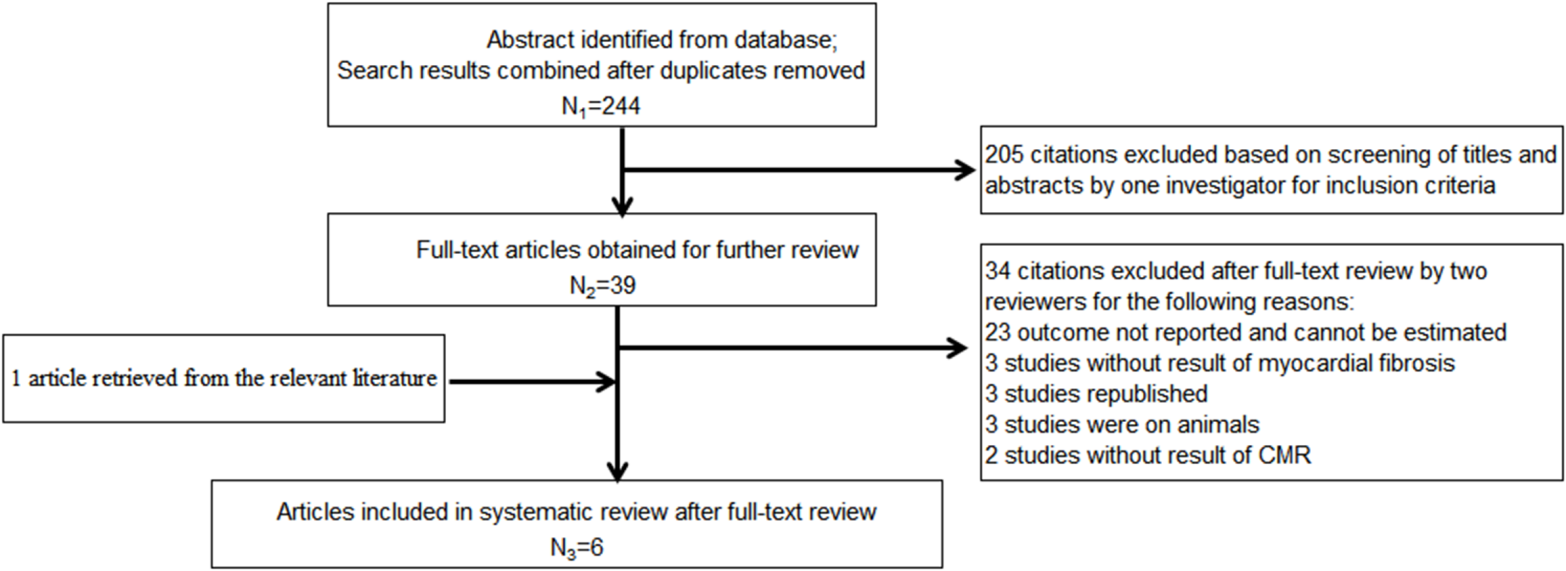
Flow chart of the systematic search process.

After retrieval from the database shown above and rechecking, 244 articles of potentially relevant studies were needed to be further classified. After screening the abstract, 39 articles were required to be read in full. Of these, five articles were eligible (16, 32–35). Another additional article was included after a hand search of the reference lists (36). Totally, there were six studies included in this meta-analysis. **Figure 1** shows the searching progress. All the six studies are published in English (16, 32–36). Except two studies from the US which were included, each of the other countries, including Australia, Canada, United Kingdom and Taiwan, China, reported one study. Totally, there are 178 patients who received empagliflozin treatment and 106 assigned to the placebo group. The sample size ranged from 17 to 40 in the empagliflozin treatment group while 8 to 40 in the control group. Four studies (32, 33, 35, 36) involved the patients with type 2 diabetes mellitus, and the remaining two (16, 34) were non-diabetic patients with HF with a reduced ejection fraction (HFrEF). Of the four studies that included type 2 diabetes mellitus, one included patients with coronary heart disease (35), and the remaining three included only a subset of patients with coronary heart disease (32, 33, 36). With regard to the left ventricular ejection fraction, two studies included patients with HF with reduced ejection fraction (16, 34). The left ventricular ejection fraction of patients in the remaining four studies were normal (32, 33, 35, 36). Empagliflozin was administered at 10 mg/day in three studies (16, 34, 35), 25 or 12.5 mg/day in one study (33), and the remaining two did not provide the detailed information (32, 36). Patients were evaluated with cardiac magnetic resonance (CMR), which was acquired in the supine position with either a 1.5-T magnet (16, 33) or a 3.0-T magnet (32, 34–36). ECV was calculated and analyzed in five studies (16, 33–36) and the native T1 value in three studies (32, 35, 36). The follow-up time was 12 weeks in one study (36) or 6 months in the remaining five studies (16, 32–35). **Table 1** summarizes the detailed characteristics of these six included studies.

**Table 1:** Characteristic of six included studies.

First author, year	Country	Characteristics of participants	Dose of empagliflozin	Number of participants, n		Mean age, year		Male (%)		Follow-up time	MR system	Outcome index
				Treatment	Control	Treatment	Control	Treatment	Control			
Cohen, N. D., 2019 (32)	Australia	T2DM	NA	17	8	63 ± 7	64 ± 9	59	75	6 months	3.0 T	Native T1 value
Hsu, J. C.,2019 (33)	Taiwan, China	T2DM	25 or 12.5 mg/day	35		64 ± 10		49		6 months	1.5 T	ECV
Santos-Gallego, C. G., 2019 (34)	USA	Non-diabetic patients with HFrEF	10 mg/day	40	40	NA	NA	NA	NA	6 months	3.0 T	ECV
Mason, T., 2021 (35)	Canada	T2DM with coronary artery disease	10 mg/day	39	35	62 ± 8	64 ± 10	87	97	6 months	3.0 T	ECV, native T1 value
Requena-Ibáñez, J. A., 2021 (16)	USA	Non-diabeitc patients with HFrEF	10 mg/day	29	23	NA	NA	NA	NA	6 months	1.5 T	ECV
Thirunavukarasu S, 2021 (36)	United Kingdom	T2DM	NA	18		67 ± 11		72		12 weeks	3.0 T	ECV, native T1 value

T2DM, type 2 diabetes mellitus; HFrEF, heart failure with reduced ejection fraction; NA, not available; MR, magnetic resonance; ECV, extracellular volume.

### Quality assessment of included studies

The quality assessment of these six studies is shown in **Table 2**. There are three articles for cohort studies (32, 33, 36), which were considered high quality if five or more scores on the NOS were achieved. All three studies were of high quality according to the NOS scoring. The remaining three studies were RCTs (16, 34, 35). According to the methodological quality evaluation of the RCT, five of the six scoring criteria of the two studies were low risk and one was unknown risk, indicating that the two studies were considered to be of high quality. The rest of the studies was considered of poor quality because of three unknown risks. Totally, although the methodological quality of one study was assessed as poor quality, we can still consider the methodological quality of the six included studies in this meta-analysis to be good.

**Table 2:** Quality assessment and risk of bias.

First author, year	Type of study design	Score of methodological quality
Cohen, N. D., 2019 (32)	Matched cohort study	9 (NOS-Cohort studies)
Hsu, J. C.,2019 (33)	Cohort study	9 (NOS-Cohort studies)
Thirunavukarasu S, 2021 (36)	Single-center prospective, longitudinal, observational cohort study	8 (NOS-Cohort studies)
Mason, T., 2021 (35)	Double-blind, randomized, placebo-controlled	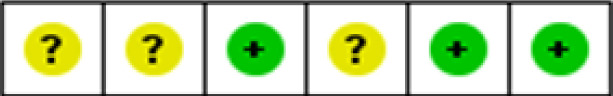	
Requena-Ibáñez, J. A., 2021 (16)	Randomized to receive either empagliflozin or matching placebo	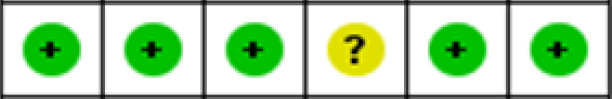	
Santos-Gallego, C. G., 2019 (34)	Randomized controlled trial	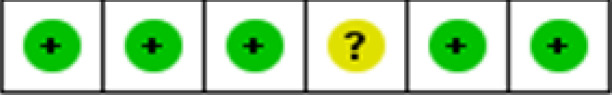	

NOS, Newcastle–Ottawa Scale risk of bias assessment: green color, low risk; yellow color, unknown risk.

### Empagliflozin and ECV

**Figure 2:**
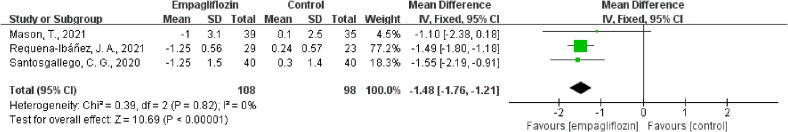
Forest plot of the △ECV.

**Figure 3:**
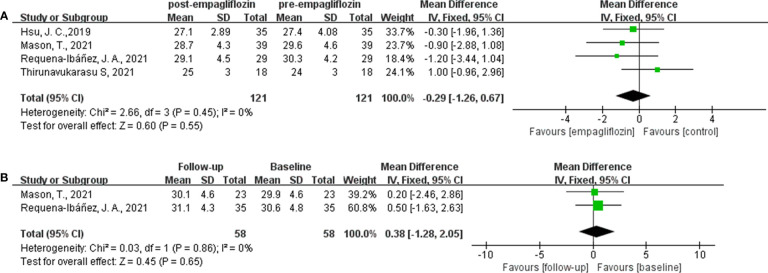
Forest plot of the ECV through a self before–after comparison. **(A)**: empagliflozin group; **(B)**: placebo group.

Of the six included studies, five studies reported the ECV as an outcome (16, 33–36). Three of these studies (16, 34, 35) reported ECV both before and after empagliflozin or placebo treatment, respectively, from which △ECV were calculated and analyzed. Additionally, the merged WMD value of △ECV by the fixed-effect model and the pooled effect size in favor of empagliflozin were -1.48 (95% CI -1.76 to -1.21, *P* < 0.00001). Heterogeneity analysis did not find any heterogeneity (chi^2^ = 0.39, *P* = 0.82, *I* ^2^ = 0%) **(** **Figure 2** **)**, which indicated that empagliflozin could significantly reduce ECV compared to placebo. At the same time, we also pooled the WMD of ECV before and after treatment in the empagliflozin group. As a result, in the treatment group, empagliflozin did not reduce ECV after treatment compared with before (WMD = -0.29, 95% CI -1.26 to 0.67, *P* = 0.55), and no heterogeneity was calculated (chi^2^ = 2.66, *P* = 0.45, *I* ^2^ = 0%) (**Figure 3A**). A further combination of the ECV values in the placebo group before and after treatment showed that placebo did not increase ECV (WMD = 0.38, 95% CI -1.28 to 2.05, *P* = 0.65). Moreover, heterogeneity analysis did not find any heterogeneity (chi^2^ = 0.03, *P* = 0.86, *I* ^2^ = 0%) (**Figure 3B**). Although we did not find that empagliflozin reduced ECV through a self before–after comparison, empagliflozin had a tendency to reduce ECV compared to the trend of increasing ECV in the placebo group. Totally, empagliflozin has the effect of reducing ECV.

### Native T1 value

**Figure 4:**
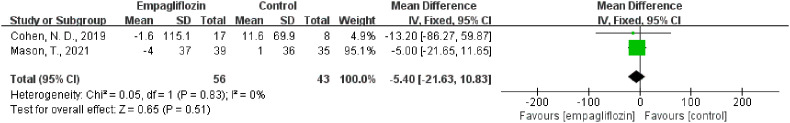
Forest plot of the △native T1 value.

**Figure 5:**
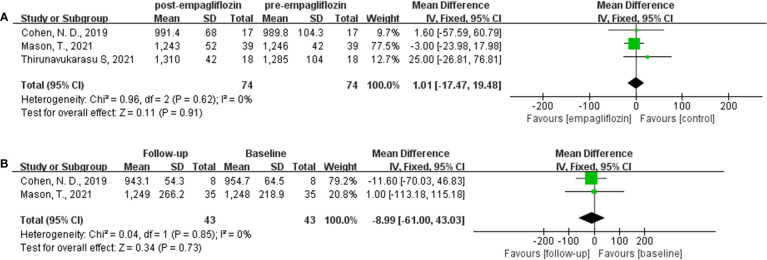
Forest plot of the native T1 value through a self before–after comparison. **(A)**: empagliflozin group; **(B)**: placebo group.

Three studies analyzed the native T1 value (32, 35, 36). Two studies (32, 35) reported the native T1 value both before and after empagliflozin or placebo treatment, respectively, from which △native T1 values were analyzed. The fixed-effect model was used to merge the WMD values of the △native T1 value, meanwhile the pooled effect size showed no statistical difference between empagliflozin and placebo groups (WMD = -5.40, 95% CI -21.63 to 10.83, *P* = 0.51). Heterogeneity analysis showed that no heterogeneity exists (chi^2^ = 0.05, *P* = 0.83, *I* ^2^ = 0%) **(** **Figure 4** **)**. This means that empagliflozin had no effect on reducing the native T1 value when compared to placebo. Further analyses combined the WMD of native T1 value in self before–after comparisons and also found the same result (empagliflozin group: WMD = 1.01, 95% CI -17.47 to 19.48, *P* = 0.91, heterozygosity test, chi^2^ = 0.96, *P* = 0.62, *I* ^2^ = 0%; placebo group: WMD = -8.99, 95% CI -61.00 to 43.03, *P* = 0.73, heterozygosity test, chi^2^ = 0.04, *P* = 0.85, *I* ^2^ = 0%) (**Figure 5**). In summary, empagliflozin could not reduce the native T1 value.

### Publication bias

**Figure 6:**
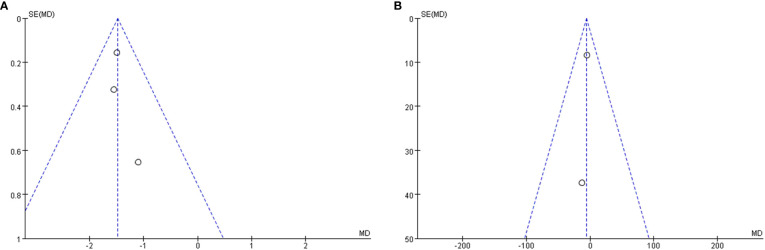
Funnel plot of publication bias. **(A)**: △ECV; **(B)**: △native T1 value.

A funnel plot was done to show the publication bias, and the results are shown in **Figure 6**. Due to the limited number of included studies, selection bias is significant but inevitable.

## Discussion

This meta-analysis summarized the effect of empagliflozin on diffuse myocardial fibrosis using CMR T1 mapping and conformed the association of empagliflozin with various cardiovascular benefits by reducing diffuse myocardial fibrosis. ECV could act as a non-invasive imaging tool to assess diffuse myocardial fibrosis and monitor disease progression.

As a kind of antidiabetic drug, SGLT2 inhibitors are effective by blocking glucose reabsorption in the proximal convoluted tubule of the kidney. In addition to the hypoglycemic effects, SGLT-2 inhibitors have been demonstrated to reduce the risk of cardiovascular outcomes and mortality in both clinical trials and animal studies (16, 20, 21, 32–36). Therefore, the 2021 European Society of Cardiology (ESC) guidelines recommended SGLT-2 inhibitors as first-line treatment for patients with HFrEF (37). Similarly, the Canadian Cardiovascular Society (CCS) and the Canadian Society for Heart Failure (CHFS) have updated their 2017 guidelines for the management of HF, including recommendations for the use of SGLT2 inhibitors in patients with and without HF with T2DM (38). All of the above indicated the important role of SGLT2 inhibitors in cardioprotective effects. Due to the rapid manifestation of cardiovascular benefit, Cowie MR et al. proposed an opinion that the cardiovascular benefits of SGLT2 inhibitors may be beyond glycemic control (39). For instance, increased urine sodium, reduced plasma volume, improved vascular resistance, reduced blood pressure and so on might account for the rapid cardioprotective effects. Meanwhile, Muscelli E et al. also proposed that the beneficial properties of empagliflozin include direct and indirect action on cardiac (40). Quagliariello V et al. studied the effect of empagliflozin on myocardial strain in non-diabetic mice treated with doxorubicin and found that empagliflozin significantly improves cardiac function by participating in NLRP3- and MyD88-related pathways and reducing adriamycin-treated iron cell apoptosis, fibrosis, and inflammation in mice (20). Lee TM et al. reported that dapagliflozin could attenuate cardiac fibrosis in postinfarcted rats (21). That is, the effect on myocardial fibrosis might be the reason why SGLT2 inhibitors could reduce the incidence of cardiovascular events.

Myocardial fibrosis, as a common pathological feature of various cardiac diseases, could be divided into two types (1, 30, 41). The first one is focal myocardial fibrosis, characterized by the macroscopic focal fibrotic scar in the myocardium. With its irreversible properties, it often presents in the terminal stages of HF and acute ischemic condition. The second type is diffuse myocardial fibrosis, characterized by perivascular deposition of fibrotic tissue and diffuse interstitial lung disease (1) in nearly all chronic cardiac diseases (2–5). Compared with focal myocardial fibrosis, diffuse myocardial fibrosis is a gradual process rather than induced by cell death. It is potentially reversible if the disease progression is well controlled; in turn, if the condition worsens, myocyte apoptosis and irreversible replacement fibrosis might follow (6, 12, 13). As its pathological changes, diffuse myocardial fibrosis could result in adverse left ventricular remodeling, increased myocardial stiffness, and even diastolic dysfunction (6). Therefore, diffuse myocardial fibrosis plays a unique role in the development of HF and leads to unfavorable outcomes in patients with HF, especially in elderly people (7–10). In this regard, it is essential to understand the development and clinical consequences of diffuse myocardial fibrosis from a prognostic perspective (1).

Currently, the gold standard for diagnosis of myocardial fibrosis is myocardial biopsy (42). However, its application is limited because of sampling errors, invasive operation, and un-quantification. Besides, in the process of clinical diagnosis and treatment, it is impossible to perform myocardial biopsy for every patient. Therefore, selecting a non-invasive diagnostic technique is of great clinical significance to evaluate the therapeutic effect of SGLT2 inhibitors on myocardial fibrosis. However, CMR using T1 mapping to characterize myocardial tissue has the potential capability to measure diffuse myocardial fibrosis without invasion (24–26). Native T1, the value of T1 in the absence of a gadolinium contrast agent, is influenced by both intracellular and extracellular factors (43, 44). As such, native T1 would seem less sensitive to interstitial changes in diffuse myocardial fibrosis compared with ECV, which might be the reason why empagliflozin could not reduce the native T1 values in our meta-analysis. However, differences in the study types (RCTs and cohorts) may be one of the reasons for the differences in results of ECV and T1. Particularly in patients with renal dysfunction, native T1 could serve as an alternative evaluation for cardiac diseases without the need for a gadolinium contrast agent. ECV was calculated by native and post-contrast T1 mapping techniques and adjusted to the hematocrit (28), non-invasively measuring the percentage of extracellular myocardium volume. Moreover, several studies have shown that the value of ECV in patients with HF is directly related to collagen deposition (45). Unfortunately, a large study conducted by Treibel et al. did not find a relationship between the ECV and collagen deposition in aortic stenosis patients (3). In addition, ECV would be overvalued by confounding factors including myocardial edema and/or inflammation, infiltration, and ischemia considering the inability of identifying fibrous tissue directly by measuring the total interstitial space (29, 30). Despite the limitations, ECV is reproducible and could act as an excellent non-invasive imaging tool to diffuse myocardial fibrosis (31). In our meta-analysis, empagliflozin could significantly reduce ECV compared to placebo, indicating the cardiovascular benefits of empagliflozin by reducing diffuse myocardial fibrosis in the myocardium. We did not find that empagliflozin reduced ECV through a self before–after comparison, while a tendency to reduce ECV was observed in the empagliflozin group compared to the trend of increasing ECV in the placebo group. Totally, empagliflozin has the effect of reducing diffuse myocardial fibrosis.

Currently, there are few studies concerning the mechanism of SGLT2 inhibitors on myocardial fibrosis. Lee et al. proposed a new mechanism of dapagliflozin to reduce myocardial fibrosis by mediating myocardial macrophage polarization through the STAT3 signaling pathway (21). The protective effect against diabetic cardiomyopathy and myocardial fibrosis of dapagliflozin was found through the suppressed endothelial-to-mesenchymal transition and fibroblast activation *via* AMPKα/TGF β/Smad signaling inT2DM rats (46). In addition, empagliflozin has also been found to reduce doxorubicin-treated fibrosis in mice by participating in NLRP3- and MyD88-related pathways (20). Empagliflozin inhibited fibrosis by inhibiting the TGF β/Smad pathway and activating the Nrf2/ARE signaling pathway, as also reported by (17). Notably, inhibition of autophagy (autophagic death) may also be an important cardioprotective mechanism of empagliflozin (47). At present, the mechanisms of SGLT2 inhibitors on myocardial fibrosis still remain elusive and more mechanisms needs to be further explored and studied.

The number of included studies was too small, and the sample size was also not large enough which could affect the results of this meta-analysis. In addition, the methodological quality of individual studies was heterogenetic. Therefore, more prospective clinical studies with larger sample sizes conducted and included to the analysis in the future may strengthen this evidence.

## Conclusions

In conclusion, our meta-analysis summarized the effect of empagliflozin on diffuse myocardial fibrosis using CMR T1 mapping and conformed the association of empagliflozin with various cardiovascular benefits by reducing diffuse myocardial fibrosis. ECV could act as a non-invasive imaging tool to assess diffuse myocardial fibrosis and monitor disease progression. In the further, more studies focusing on the mechanism still need to be done.

## Data availability statement

The raw data supporting the conclusions of this article will be made available by the authors, without undue reservation.

## Author contributions

HW, JZ and LL designed the study and wrote the manuscript. HW, LD and LT performed the literature searches and collected the data. HW, LD and YT performed the statistical analysis. All authors approved the final content of the manuscript.

## Funding

This work was supported by the Shandong Provincial Natural Science Foundation of China Grants [ZR2020QH266], the Imaging Research Fund of LunQin [SD-202008-011], and the Science and Technology Development Plan of Traditional Chinese Medicine in Shandong Province [2019-0374].

## Conflict of interest

The authors declare that the research was conducted in the absence of any commercial or financial relationships that could be construed as a potential conflict of interest.

## Publisher’s note

All claims expressed in this article are solely those of the authors and do not necessarily represent those of their affiliated organizations, or those of the publisher, the editors and the reviewers. Any product that may be evaluated in this article, or claim that may be made by its manufacturer, is not guaranteed or endorsed by the publisher.
